# Supplementary figures and images for: Extensible membrane nanotubules mediate attachment of Trypanosoma cruzi epimastigotes under flow
Source: PLoS One. 2023 Mar 22;18(3):e0283182. doi: 10.1371/journal.pone.0283182 (PMC10032539; doi:10.1371/journal.pone.0283182)

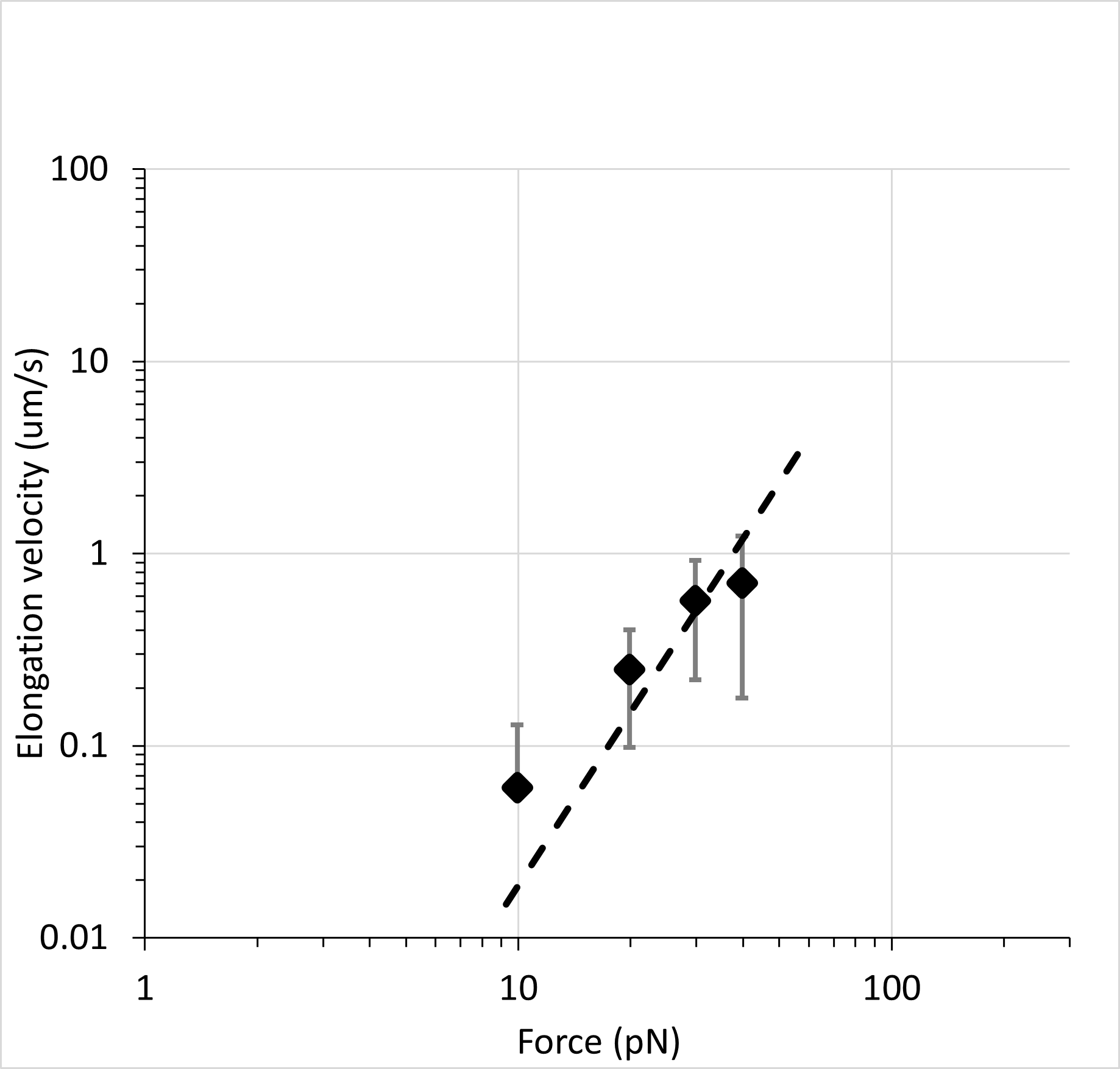

Supplement: S1 Fig — The line is a fit using the model by Brochard-Wyart et al. [25] and results in an estimate of surface viscosity of the flagellar membrane of Trypanosoma cruzi at ~50 10−6 Pa∙s∙m, consistent with the properties of cell membranes from other types of cells. (TIF) [file pone.0283182.s001.tif]

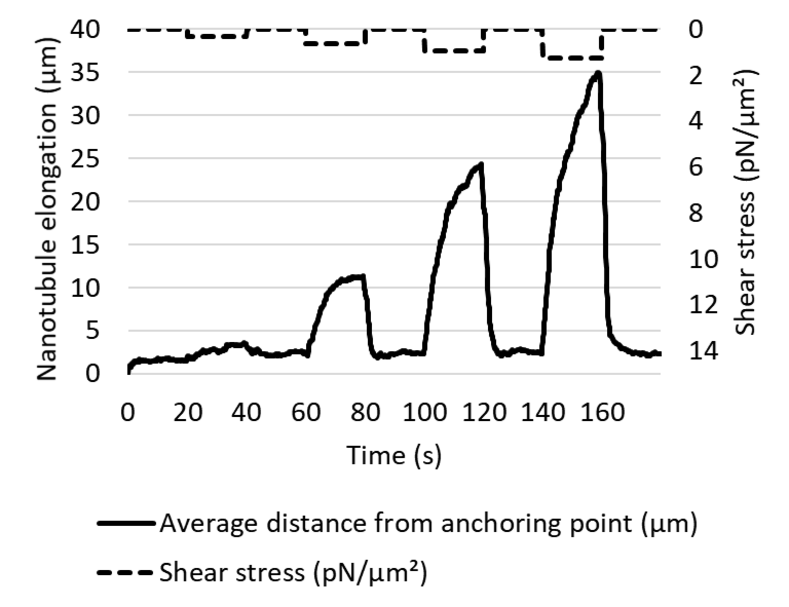

Supplement: S2 Fig — Each epimastigote was subjected to a shear stress cycle of 0.31 –– 0.62–0.93–1.24 pN/um2; each pulse was 20 s long and followed by a 20 s pause. There was an initial pause 20 s long. The curve shows the average elongation of 12 different parasites. Note both elongation regimes: a fast extension and a creep regime. Differences between epimastigotes were mainly related to the maximum length reached at each shear stress. (TIF) [file pone.0283182.s002.tif]

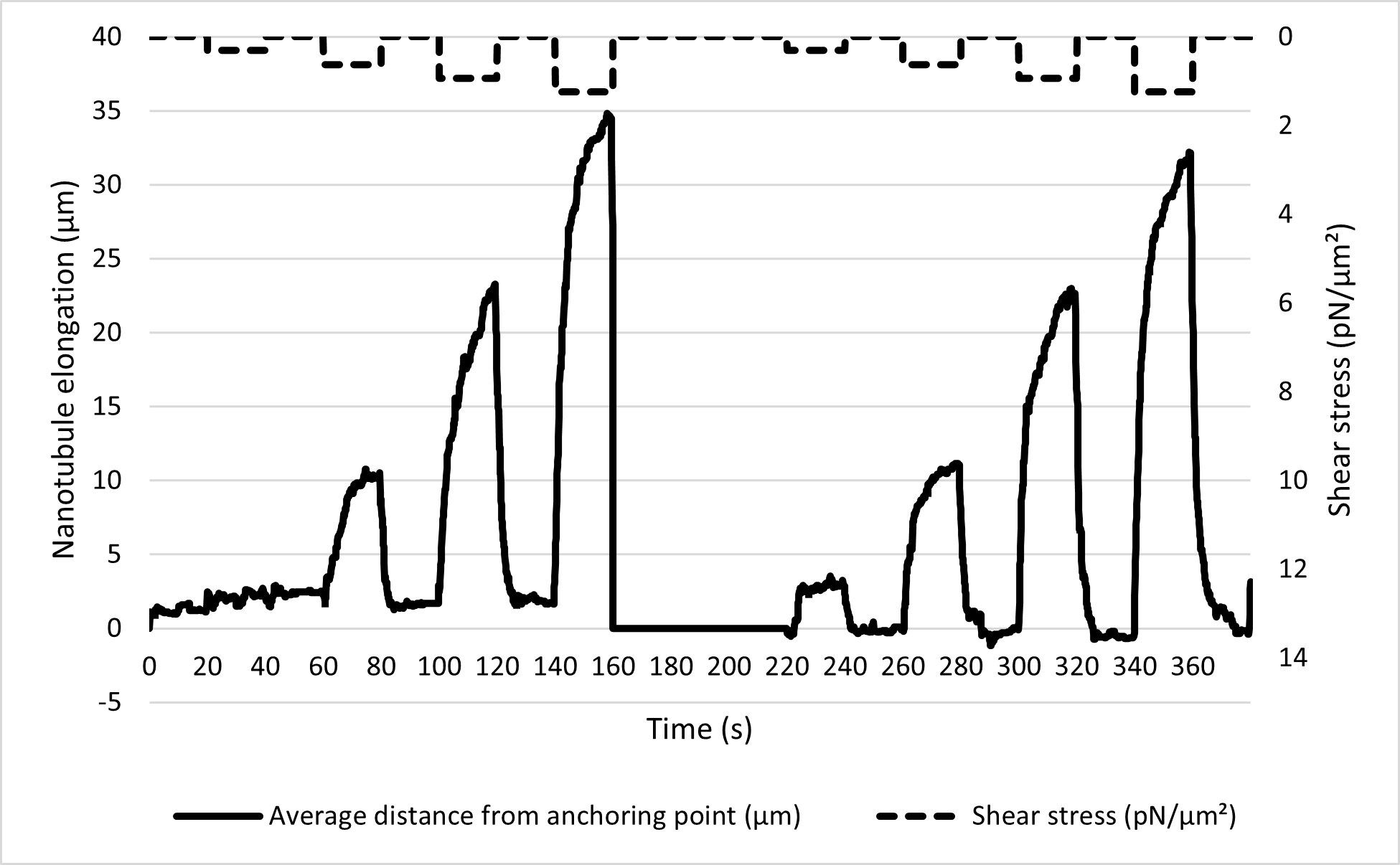

Supplement: S3 Fig — Each epimastigote was subjected to two shear stress cycles of 0.31 –– 0.62–0.93–1.24 pN/um2; each pulse was 20 s long and followed by a 20 s pause. There was a pause of 1 min between cycles. The filament length during this pause was averaged and set to 0, assuming this as the initial anchoring point for the second cycle. The curve shows the average elongation of five different parasites. There is a subtle reduction in maximum length reached in the second cycle. (TIF) [file pone.0283182.s003.tif]

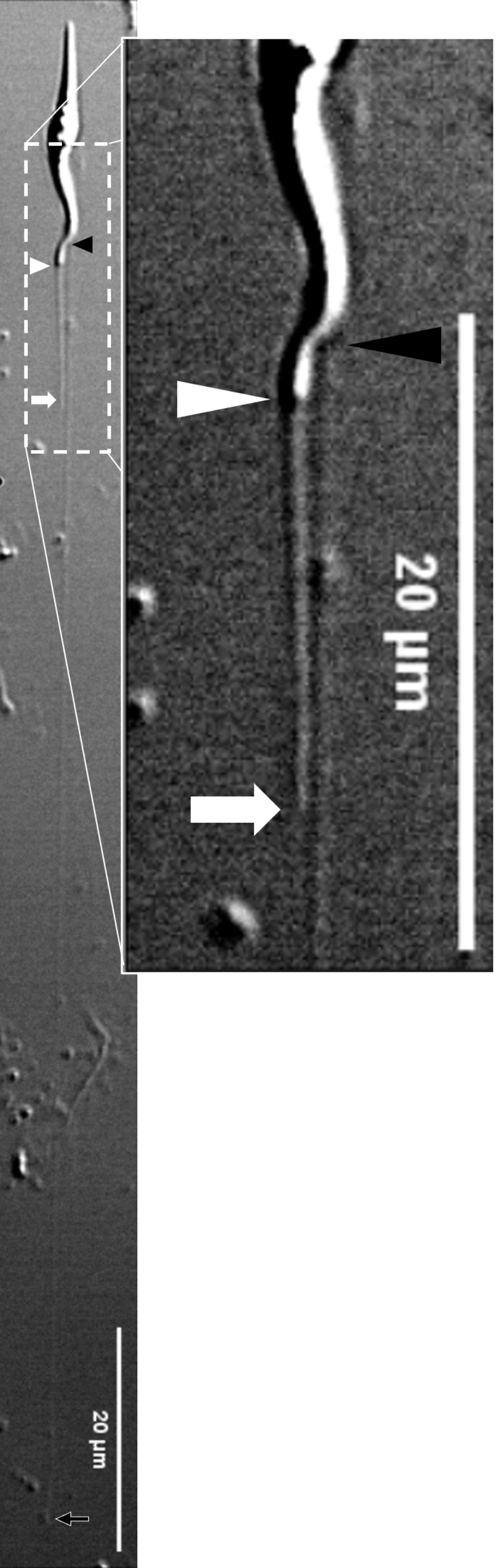

Supplement: S4 Fig — Illustrative multiply tethered epimastigote, observed at a shear stress of 1.55 pN/μm2. The anchoring point of the first adhered filament is marked with the black arrow. Note how far the filament extends without rupturing or detaching. The second filament emerged during the extension of the first one, its anchoring point is marked by a white arrow. The inset shows the parasite-end of the filaments (black arrow head for the first filament and white arrowheat for the second), note the diferent location of each one. (TIF) [file pone.0283182.s004.tif]
