# Supplementary material for: Extensible membrane nanotubules mediate attachment of Trypanosoma cruzi epimastigotes under flow
Source: PLoS One. 2023 Mar 22;18(3):e0283182. doi: 10.1371/journal.pone.0283182 (PMC10032539; doi:10.1371/journal.pone.0283182)
Supplement: Attachment — Submitted filename: 20221212 Rebuttal Plos Pathogens.pdf [file pone.0283182.s010.pdf]

Dear editors,

We thank the reviewers of PLoS Pathogens for their comments on our paper. In comparison to our first submission, we have changed part of the scope and analysis of our manuscript. Then the data now presented focusses on the biophysical properties of the filaments found in T. cruzi parasites.

We have answered the initial inquiries that the editors had then:

Q/ Following our discussions, we felt that filament formation is potentially important in vivo, but that it remains unclear whether the filaments observed indeed reflect a mechanism of attachment in vivo or whether shear stress produces filaments in vivo.
R/ Although we currently have no data to prove this is the case, we re-wrote the paper and performed additional data analysis to focus on the biophysical properties of the filaments. As such we believe PLoS One may be a better fit due to its more general audience and focus.

Q/ Other comments related to a relative lack of quantification to provide evidence that the examples shown are representative.
R/ We further analyzed the data and added more details on the number of observations. Since we have changed part of our analysis and scope, we now have more evidence and information that could be of interest to PLoS One readers. The additional quantitative analysis of the filaments shows that the biophysical properties are consistent with those for other cells as reported in the literature.

Q/ Finally, it was noted that continued motility following attachment via the terminal part of the flagellum has been reported previously (in ref. 19).
R/ We also see parasites that continue to move after attachment. However, in the manuscript we make a finer point in that this movement continues when there is no flow and a single filament, whereas parasites attached by multiple filaments or under flow reduce their motility.

We now believe that the manuscript is suitable to be published in PLoS one.

Sincerely,

The authors
