# Supplementary material for: Extensible membrane nanotubules mediate attachment of Trypanosoma cruzi epimastigotes under flow
Source: PLoS One. 2023 Mar 22;18(3):e0283182. doi: 10.1371/journal.pone.0283182 (PMC10032539; doi:10.1371/journal.pone.0283182)
Supplement: Attachment — Submitted filename: Response to Reviwers 20230225.docx [file pone.0283182.s011.docx]

Response to the editor’s comments:

1. Please ensure that your manuscript meets PLOS ONE's style requirements, including those for file naming. The PLOS ONE style templates can be found at https://journals.plos.org/plosone/s/file?id=wjVg/PLOSOne_formatting_sample_main_body.pdf and <https://journals.plos.org/plosone/s/file?id=ba62/PLOSOne_formatting_sample_title_authors_affiliations.pdf>.

Response: We have revised our manuscript in order to fulfill the journal’s requirements

2. We note that the grant information you provided in the ‘Funding Information’ and ‘Financial Disclosure’ sections do not match.

When you resubmit, please ensure that you provide the correct grant numbers for the awards you received for your study in the ‘Funding Information’ section.

Response: The funding we received was not in the form of formal grants, and there are no grant numbers. We included the (intramural) financial support in the acknowledgements.

3. We note that you have indicated that data from this study are available upon request. PLOS only allows data to be available upon request if there are legal or ethical restrictions on sharing data publicly. For more information on unacceptable data access restrictions, please see <http://journals.plos.org/plosone/s/data-availability#loc-unacceptable-data-access-restrictions>.

b) If there are no restrictions, please upload the minimal anonymized data set necessary to replicate your study findings as either Supporting Information files or to a stable, public repository and provide us with the relevant URLs, DOIs, or accession numbers. For a list of acceptable repositories, please see <http://journals.plos.org/plosone/s/data-availability#loc-recommended-repositories.te>

Response: we have uploaded the data which we analyzed as a dataset and the movies to Figshare. You can find them in:

supporting information: https://doi.org/10.6084/m9.figshare.22178795

S1 Dataset: https://doi.org/10.6084/m9.figshare.22178789.v1

S1 Movie: https://doi.org/10.6084/m9.figshare.22177418.v1

S2 Movie: https://doi.org/10.6084/m9.figshare.22177583.v1

S3 Movie: https://doi.org/10.6084/m9.figshare.22177586.v1

4. We note that you have included the phrase “data not shown” in your manuscript. Unfortunately, this does not meet our data sharing requirements. PLOS does not permit references to inaccessible data. We require that authors provide all relevant data within the paper, Supporting Information files, or in an acceptable, public repository. Please add a citation to support this phrase or upload the data that corresponds with these findings to a stable repository (such as Figshare or Dryad) and provide and URLs, DOIs, or accession numbers that may be used to access these data. Or, if the data are not a core part of the research being presented in your study, we ask that you remove the phrase that refers to these data.

Response: In that particular section we are describing the biophysical properties of adhesion nanotubules in T. cruzi epimastigotes after they were exposed to shear stress. We observed that some epimastigotes did not produce nanotubules, so we did not consider those parasites for the analysis of nanotubule properties, even if we observed them, so the phrase “data not shown” was inaccurate. We have rewritten lines 379 – 385 to clarify that.

5. Please review your reference list to ensure that it is complete and correct. If you have cited papers that have been retracted, please include the rationale for doing so in the manuscript text, or remove these references and replace them with relevant current references. Any changes to the reference list should be mentioned in the rebuttal letter that accompanies your revised manuscript. If you need to cite a retracted article, indicate the article’s retracted status in the References list and also include a citation and full reference for the retraction notice.

Response:

Concerning the articles we referenced in our manuscript, we could not find retracted articles. We did find one paper that has a corrected section that is not related to our study or the data we cited. Nevertheless, taking into consideration a comment given by one of the reviewers (below), we have shortened the first part of the introduction, the section in which we used the mentioned article, and we have removed that reference from our manuscript.

On the other hand, we have added references 8 and 9 to broaden the description of the flagellum functionalities in the introduction.

(Removed) Ballesteros-Rodea G, Santillán M, Martínez-Calvillo S, Manning-Cela R. Flagellar Motility of Trypanosoma cruzi Epimastigotes. Journal of Biomedicine and Biotechnology. 2012

8. Vaughan S. Assembly of the flagellum and its role in cell morphogenesis in Trypanosoma brucei. Curr Opin Microbiol. 2010 Aug;13(4):453–8.

9. Sharma R, Peacock L, Gluenz E, Gull K, Gibson W, Carrington M. Asymmetric Cell Division as a Route to Reduction in Cell Length and Change in Cell Morphology in Trypanosomes. Protist. 2008 Jan;159(1):137–51.

Response to de reviewers

- Reviewer #1, Dr. Rodrigo Pedro Soares

1. I think the text (lines 1-55) could be shortened or even removed since it includes basic information on T. cruzi biology. Introduction could start at the 3rd paragraph since the flagellum is the main focus of the paper.

Response: We thank the reviewer for the comment, and agree the introduction was a bit lengthy. We have rewritten the introduction to improve its focus and clarity.

2. Line 425 - I do not think is the GPI-mucins, it is probably another major glycoconjugate, the GIPLs. The best reference to cite here is: Trypanosoma cruzi: involvement of glycoinositolphospholipids in the attachment to the luminal midgut surface of Rhodnius prolixus.

Nogueira NF, Gonzalez MS, Gomes JE, de Souza W, Garcia ES, Azambuja P, Nohara LL, Almeida IC, Zingales B, Colli W.

Exp Parasitol. 2007 Jun;116(2):120-8. doi: 10.1016/j.exppara.2006.12.014.

Response: We agree that the proteins involved in the process could indeed be glycoinositolphospholipids (GIPL) and the article suggested is very interesting; considering that the focus of the manuscript is on the biomechanical properties of membrane nanotubules, we have removed assumptions regarding the molecules that could mediate attachment.

Minor comments.

a. line 195 -– nanotubules Corrected
b. lines 325, 336/337 and 370/399 - 1,55/1,24 or 1.55/1.24? We corrected all numbers to period separated decimals instead of comma separated decimals.
c. Lines 361-362/396 and several parts in figure legends - 0.31 –– 0.62 – 0.93 – 1.24 corrected
d. line 460 - total? Corrected
e. line 473 - brucei not Brucei ✓	Corrected
- Reviewer #2, Dr. David Sacks
  - The submitted manuscript has been transferred from Plos Pathogens following revisions to address the main comments of the reviewers. The revised manuscript is well written and it adequately addresses the concerns regarding the relevance of the filaments to attachment in vivo, and the reproducibility of the observations regarding filament elongation under varying levels of shear stress. Despite the absence of in vivo studies, the data on on the biophysical properties of the filaments are sound, they are novel with respect to T. cruzi, and provide a meaningful advance to the study of microbial adhesion under conditions of shear stress.

Response: We appreciate the time taken to review and comment the paper.

- Reviewer #3, Dr. Sergio Schenkman

1. It should be discussed that attachment only occurs in parasite washed in PBS (why?) and extended in PBS.

Response: We thank the reviewer for the comment. We used PBS because it has a known and stable viscosity (necessary to calculate shear stress and the resulting forces on the parasite), is isoosmolar; then, we did not disturb the homeostasis or motility of the parasite. Attachment of the parasites was only studied under PBS, but is likely to happen in other buffers.

2. The nanotubes can be seen after addition of medium and shear force (I know that they remain attached)?

Response: Parasites were added in the medium, and nanotubules were observed after these attached to the surface and force resulting from shear stress was turned on. We did not change media for the reasons explained above.

3. The authors should clarify what surface they used? Only glass?

Response: We used non-functionalized glass, and have clarified this in the methods, we thank the reviewer for pointing this oversight. We also tested poly-l-lysine but found it was not necessary to study the mechanical properties of the nanotubules and resulted in additional complexity.

4. The text should include and discuss the formation of similar structures in T. cruzi amastigotes (J . Euk. Microbioi.,43(4)1996, 275-28 and references in).

Response: We thank the reviewer for pointing it out this paper as we had missed it. We now discuss it in the context of our findings in lines 512 - 526. Similarly, we included reference 59 to enrich the discussion.

5. The author suggest that T. cruzi mucins could be in the membrane of the nanotubes. It would be interesting if they test it using monoclonal antibodies. There are also antibodies that labeled the flagellum membrane (FCBP for example) that could be used, if possible.

Response: We agree that this would be an interesting experiment to attempt in order to elucidate the molecules involved in this attachment process. However, considering that the scope of our research is to describe the biomechanical properties of the nanotubules in the attachment process, not to identify the molecules that mediate attachment, we removed the part where we discuss the molecules that could mediate the interaction since it is detract from the focus of the paper.

6. Recheck orthography. I found a few typos

Response: We corrected a couple typos we found using our text editing software, hopefully they are the same.

Again, we thank the reviewers for taking the time to review the paper and make constructive comments that have improved the quality of the paper.
